# Modelling the effect of a dengue vaccine on reducing the evolution of resistance against antibiotic due to misuse in dengue cases

**DOI:** 10.1186/s12976-020-00125-8

**Published:** 2020-05-13

**Authors:** Ana Kurauchi, Claudio Jose Struchiner, Annelies Wilder-Smith, Eduardo Massad

**Affiliations:** 1 School of Medicine, University of Sao Paulo, Sao Paulo, Brazil; 2 School of Applied Mathematics, Fundacao Getulio Vargas, Rua Praia de Botafogo 190, Rio de Janeiro, CEP - 22250-900 Brazil; 3 Department of Disease Control, London School of Hygiene and Tropical Medicine, London, UK; 4 Heidelberg Institute of Global Health, University of Heidelberg, Heidelber, Germany

**Keywords:** Dengue, Antibiotic resistance, Vaccine, Mathematical models

## Abstract

**Background:**

This paper intends to check whether and how a hypothetical dengue vaccine could contribute to issue of evolution of bacteria resistance against antibiotics by reducing the number of patients that would inappropriately being treated with antibiotics.

**Methods:**

We use a new mathematical model that combines, in a novel way, two previously published papers, one on the evolution of resistance against antibiotics and one classical Ross-Macdonald model for dengue transmission.

**Results:**

The model is simulated numerically and reproduces a real case of evolution of resistance against antibiotics. In addition the model shows that the use of a hypothetical dengue vaccine could help to curb the evolution of resistance against an antibiotic inappropriately used in dengue patients. Both the increase in the proportion of resistant bacteria due to the misuse of antibiotics in dengue cases as a function of the fraction of treated patients and the reduction of that proportion as a function of vaccination coverage occur in a highly non-linear fashion.

**Conclusion:**

The use of a dengue vaccine is helpful in reducing the rate of evolution of antibiotic resistance in a scenario of misuse of the antibiotics in dengue patients.

## Background

Antibiotics are one of the major breakthroughs in the history of medicine and have saved millions of lives [1]. However, its misuse or overuse can also have disastrous consequences [2]. Overuse is frequent: approximately two-third (68%) of antibiotics are prescribed for upper respiratory infections (URTIs) [3, 4] but > 80% of such prescriptions have been found to be unnecessary and inappropriate with adverse outcome including the menace of antibiotic resistance.

Antibiotic resistance (the ability of microbes to evolve and withstand the effects of antibiotics) is a significant cause of morbidity and mortality globally [5–7], and antibiotic over-consumption is the main driver of antibiotic resistance [8]. The association between antibiotic consumption and resistance is well documented across spatial and temporal scales at individual hospitals [9], nursing homes [10], primary care facilities [11], and communities [12], as well as across countries [13].

Bacteria have become resistant to antimicrobial agents as a result of chromosomal changes or the exchange of genetical material via plasmids and transposons [14].

Many countries have adopted national action plans on antimicrobial resistance (AMR) that aim to reduce per capita antibiotic consumption. The Global Action Plan on Antimicrobial Resistance endorsed by the member states of the World Health Organization (WHO) and affirmed at the high-level meeting on antimicrobial resistance during the 71st General Assembly of the United Nations [15], recommends that all countries collect and report antibiotic consumption data [16]. Surveillance data on country-level antibiotic use are needed to monitor national and global trends over time; compare antibiotic use among countries; provide a baseline for the evaluation of future efforts to reduce antibiotic use; enable epidemiological analysis of the association between antibiotic use and resistance over time [17, 18]; and support policies that aim to reduce antibiotic resistance.

Making better use of existing vaccines and developing new vaccines are important ways to tackle AMR and reduce preventable illness and deaths.

Dengue is a mosquito-borne infection, transmitted by the *Aedes aegypti* mosquitoes, characterized by sudden onset of fever and severe headache; resulting in shock and hemorrhage leading to death [19] in many of such patients [20, 21]. The clinical manifestations are often nonspecific, with signs and symptoms that overlap with many other febrile illnesses including bacterial infections. As a result, health care practitioners often prescribe antibiotics empirically, e.g. without confirming the diagnosis, which leads to unnecessary use of antibiotics.

In one particular study in India [22], of 370 confirmed dengue cases, 267 (74.6%)cases were prescribed antibiotics. A single antibiotic was prescribed to 225 cases (60.8% of all cases), 2 antibiotics to 33 dengue cases (8.9%), and 3 antibiotics to 9 (2.4%). Triple therapy antibiotics included cefotaxime in all prescriptions with cefixime, azithromycin, amoxyclav, doxycycline, and ceftriaxone in different combinations. Antibiotics given as dual therapy were ceftriaxone with doxyxycline, cefotaxime, or amoxyclav, and cefotaxime with doxycycline, cefixime, or metronidazole.

In another study in West Java, Indonesia [23], showed that there were 547 (17.8%) out of 3078 dengue patients that received antibiotics.

One of the authors (AK) found in the Clinics Hospital in São Paulo, Brazil, that among 103 confirmed dengue cases, 35 (34%) inappropriately received antibiotics.

Of course antibiotics are indicated when secondary bacterial infections are present in some dengue cases [24]. However, the problem of antibiotics misuse in dengue cases is related to misdiagnosing dengue with URTI [21]. To distinguish between the appropriate and inappropriate antibiotic use in dengue cases it is necessary to demonstrate the presence of bacterial infection, for instance, by collecting material for bacterial culture procedures [21].

Therefore, is seems that there is enough evidence of misuse of antibiotics in dengue patients. Such misuse could worsen the selective pressure that leads to the evolution of resistance against those antibiotics.

This paper intends to investigate whether and how a hypothetical dengue vaccine could contribute to issue of evolution of bacteria resistance against antibiotics by reducing the number of patients that would inappropriately being treated with antibiotics. We do so by using a new mathematical model that combines, in a novel way, two previously published papers, one on the evolution of resistance against antibiotics, illustrated by the case of resistance of *K. pneumoniae* resistance against Amikacine (this was one clear example of rapide evolution of resistance studied by one of the authors of the present paper) and one classical Ross-Macdonald model for dengue transmission.

## The model

The model combines two distinct models, one proposed previously in Massad, Yang and Lundberg [25] for the study of the evolution of antibiotic resistance, and one Ross-Macdonald type of dengue model [26], including the possibility of vaccination.

**Fig. 1:**
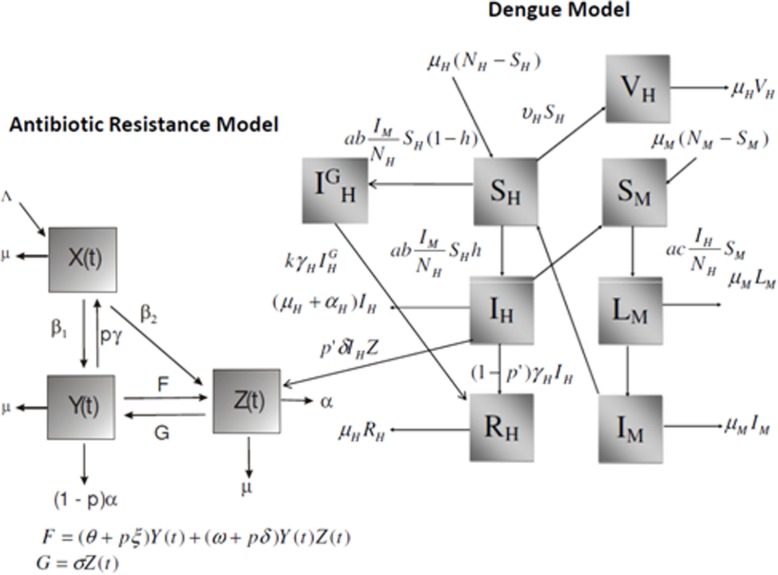
Composite model combining an antibiotic resistance model and a Ross-Macdonald dengue model with vaccination

The composite model is described in Fig. 1:

In Fig. 1, the left-hand side picture describes the model by Massad, Yang and Lundberg [25], designed to study the evolution of resistance against antibiotics. This model considered a population in a hospital environment, in which *X*(*t*) represents individuals who have been hospitalized by diverse causes with rate Λ, and are susceptible to a given infectious agent. These individuals may acquire an hospital infection by a strain of the pathogen which is sensitive to a specific antibiotic against that pathogen with a rate *β*_1_. Once infected with the sensitive strain these individuals are denoted *Y*(*t*). A fraction of *p* of those *Y*(*t*) individuals are treated with the specific antibiotic and recovers to the susceptible state again with rate *γ*.The fraction (1 − *p*) of non-treated individuals die from the infection with rate *α*. However, *Y*(*t*) individuals may be discharged from the hospital with rate *μ*. Alternatively, the susceptible individuals *X*(*t*) may acquire the infection by a strain of the pathogen which is resistant to the specific antibiotic against that pathogen with a rate *β*_2_. Once infected with the resistant strain these individuals are denoted *Z*(*t*). These individuals may either be discharged from the hospital with rate *μ* (like everyone else in the model), or die from the infection with rate *α*. The model consider the evolution of antibiotic resistance by two alternative mechanisms, one consisting in mutation, and one by plasmid transfer from the sensitive to resistant strains. These two mechanisms are represented in the figure by the composite rate *F*. The mutational component of rate *F* is described by the expression (*θ* + *pξ*)*Y*(*t*) + (*ω* + *pδ*)(*Y*(*t*)*Z*(*t*)). In this expression, *θ* is the treatment-independent mutation rate and *pξ* is the mutation rate induced by antibiotic treatment. The second component, comprises the term *ω*, which is the treatment-independent plasmid transfer and the term *pδ*, which is the plasmid transfer rate induced by antibiotic treatment (note the cross-infection term (*Y*(*t*)*Z*(*t*)). The back-mutational component of rate *G* is described by the expression *σ Z*(*t*). In this expression, *σ* is the back-mutation rate.

The right-hand side picture shown in Fig. 1 describes the Ross-Macdonald model to be used to represent dengue infection with a vaccination component. The model considers that people born with rate *μ*_*H*_ (assumed equal to the natural mortality rate) and who are susceptible to dengue are denoted *S*_*H*_(*t*). These individuals may acquire dengue infection with incidence $ab\left(\frac{I_M}{N_H}\right)$, where *a* is the mosquitoes’ biting rate, *b* is the probability of infection from mosquitoes to humans, *I*_*M*_(*t*) is the number of infected mosquitoes and *N*_*H*_ is the total human population (assumed constant by equating the birth and death rates as *μ*_*H*_). Dengue infected individuals are denoted ${I}_H^T(t)$, a fraction *h* of whom are interned in the same hospital as in the first model. Non-hospitalized dengue individuals are denoted ${I}_H^G(t)$. Alternatively the susceptible individuals may be vaccinated with rate *υ*_*H*_ and are then denoted *V*_*H*_(*t*). Hospitalized dengue-infected individuals, denoted *I*_*H*_(*t*), may either be infected with plasmids they acquire from the resistant strain infected individuals in the same hospital, *Z*_*H*_(*t*), provided that a fraction *p*' of them is mistreated with the antibiotics (note that the rate of plasmid transfer *δ* is the same), or are discharged from the hospital with rate *μ*_*H*_ (like everyone else in the model), or die from dengue infection with rate *α*_*H*_, or are infected with the sensitive strain and treated and recovered from dengue with rate *γ*_*H*_. Therefore, the term *p* ' *δI*_*H*_(*t*)*Z*(*t*) represents the plasmid transfer from *Z*_*H*_(*t*) to *I*_*H*_(*t*), by a cross-infection mechanism. Like in the classical Ross-Macdonald model, susceptible mosquitoes, denoted *S*_*M*_(*t*) may acquire dengue infection with incidence $ac\left(\frac{I_M}{N_H}\right)$, where again *a* is the mosquitoes’ biting rate, *c* is the probability of infection from humans to mosquitoes, *I*_*M*_(*t*) is the number of infected mosquitoes and *N*_*H*_ is the total human population. Once infected, these mosquitoes get into a latent state, denoted *L*_*M*_(*t*) and then either die or evolve to the infective state *I*_*M*_(*t*). Note that mosquitoes are born and die with the same rate *μ*_*M*_, which implies that the total mosquito population is assumed constant.

The model’s variables and parameters are described in Table 1.

**Table 1:** The Model’s variables, parameters, biological meaning and values

Variable/Parameter	Biological Meaning	Initial condition/Value used in the simulations
**Antibiotic Resistance Model**^**a**^		
*X*(*t*)	Individuals susceptible to the hospital infection	100
*Y*(*t*)	Individuals infected with a sensitive strain	1
*Z*(*t*)	Individuals infected with a resistant strain	0.41
Λ	Internment rate	3.33 × 10^− 2^ days^− 1^
*μ*	Discharging rate	3.33 × 10^− 2^ days^− 1^
*β*_1_	Rate of infection with the sensitive strain	6.45 × 10^− 3^ days^− 1^
*β*_2_	Rate of infection with the resistant strain	6.00 × 10^− 3^ days^− 1^
*p*	Fraction treated with the antibiotic	0.7
*γ*	Recovered from infection with the sensitive strain	2.50 × 10^− 1^ days^− 1^
*α*	Mortality rate induced by the infection	1.15 × 10^− 1^ days^− 1^
*θ*	Rate of mutation from sensitive to resistant strains	1.00 × 10^− 7^ days^− 1^
*ξ*	Treatment induced mutation rate	1.00 × 10^− 6^ days^− 1^
*ω*	Rate of spontaneous plasmids transfer	1.15 × 10^− 5^ days^− 1^
*δ*	Rate of treatment induced plasmids transfer	1.15 × 10^− 4^ days^− 1^
*σ*	Rate of back mutation from resistant to sensitive strains	1.00 × 10^− 8^ days^− 1^
**Dengue Model**^**b**^		
*S*_*H*_(*t*)	Individuals susceptible to dengue	1.00 × 10^5^
${I}_H^G(t)$	Non-hospitalized individuals infected with dengue	1.0
*I*_*H*_(*t*)	Hospitalized individuals infected with dengue	0.0
*R*_*H*_(*t*)	Individuals recovered from dengue	0.0
*V*_*H*_(*t*)	Individuals vaccinated against dengue	0.0
*S*_*M*_(*t*)	Mosquitoes susceptible to dengue	1.50 × 10^5^
*L*_*M*_(*t*)	Mosquitoes latent with dengue	1
*I*_*M*_(*t*)	Mosquitoes infective with dengue	0
*μ*_*H*_	Birth/Mortality rate	3.92 × 10^− 5^ days^− 1^
*υ*_*H*_	Vaccination rate	Variable
*a*	Mosquitoes’ biting rate	10 days^− 1^
*b*	Probability of infection from mosquitoes to humans	0.6
*c*	Probability of infection from humans to mosquitoes	0.6
*γ*_*H*_	Recovered from infection with the sensitive strain	5.0 × 10^− 1^ days^− 1^
*α*_*H*_	Dengue-induced mortality rate	1.00 × 10^−5^ days^− 1^
*p*'	Fraction of dengue-infected individuals mistreated with antibiotics.	Variable
*h*	Fraction of dengue-infected individuals that are hospitalized	0.3
*κ*	Fraction of non-hospitalized dengue-infected individuals that recover from infection	0.7
*d*	Rate of hospital discharge of dengue patients	1.00 × 10^−2^ days^−1^

^a^Parameters’ and initial conditions’ values from reference [25]

^b^Parameters’ and initial conditions’ values from reference [26]

The model is described by the following system of equations:

1 $$ {\displaystyle \begin{array}{l}\mathrm{Dengue}\ \mathrm{Model}\\ {}\\ {}\frac{dS_H(t)}{dt}=- ab\frac{I_M(t)}{N_H}{S}_H(t)+{\mu}_H\left[{N}_H-{S}_H(t)\right]-{\upsilon}_H{S}_H(t)\\ {}\frac{dI_H^G}{dt}= ab\frac{I_M(t)}{N_H}{S}_H(t)\left(1-h\right)-\left({\mu}_H+{\alpha}_H\right){I}_H^G(t)-k{\gamma}_H{I}_H^G(t)\\ {}\frac{dI_H(t)}{dt}= ab\frac{I_M(t)}{N_H}{S}_H(t)h-\left({\mu}_H+{\alpha}_H+d\right){I}_H(t)-\left(1-p\hbox{'}\right){\gamma}_H{I}_H(t)-p\hbox{'}\delta {I}_H(t)Z(t)\\ {}\frac{dR_H(t)}{dt}=\left(1-p\hbox{'}\right){\gamma}_H{I}_H(t)+k{\gamma}_H{I}_H^G(t)-{\mu}_H{R}_H(t)\\ {}\frac{dV_H(t)}{dt}={\upsilon}_H{S}_H(t)-{\mu}_H{V}_H(t)\\ {}\\ {}{I}_H^T(t)={I}_H^G(t)+{I}_H(t)\\ {}{N}_H={S}_H+{I}_H+{R}_H+{V}_H\\ {}\\ {}{Vac}_{\mathrm{cov}}(t)=\frac{1}{N_H}\underset{0}{\overset{t}{\int }}{\upsilon}_H{S}_H(s) ds\\ {}\\ {}\frac{dS_M(t)}{dt}=- ac\frac{I_H^T(t)}{N_H}{S}_M(t)+{\mu}_M\left({N}_M-{S}_M(t)\right)\\ {}\frac{dL_M(t)}{dt}= ac\frac{I_H^T(t)}{N_H}{S}_M(t)-\left({\gamma}_M+{\mu}_M\right){L}_M(t)\\ {}\frac{dI_M(t)}{dt}={\gamma}_M{L}_M(t)-{\mu}_M{I}_M(t)\\ {}\\ {}{N}_M={S}_M+{L}_M+{I}_M\\ {}\\ {}\mathrm{Antiobtic}\ \mathrm{Resistance}\ \mathrm{Model}\\ {}\\ {}\frac{dX(t)}{dt}=-{\beta}_1X(t)Y(t)-{\beta}_2X(t)Z(t)-\mu X(t)+ p\gamma Y(t)+\Lambda \\ {}\frac{dY(t)}{dt}={\beta}_1X(t)Y(t)-\left[\left(1-p\right)\alpha + p\gamma \right]Y(t)-\mu Y(t)-F\left[Y(t),Z(t)\right]+G\left[Z(t)\right]\\ {}\frac{dZ(t)}{dt}={\beta}_2X(t)Z(t)-\alpha Z(t)+F\left[Y(t),Z(t)\right]-\mu Z(t)-G\left[Z(t)\right]+p\hbox{'}\delta {I}_H(t)Z(t)\\ {}\\ {}N=X+Y+Z\\ {}F\left[Y(t),Z(t)\right]=\left(\theta + p\xi \right)Y(t)+\left(\omega + p\delta \right)Y(t)Z(t)\\ {}G\left[Z(t)\right]=\sigma Z(t)\\ {}\\ {} resis(t)=\frac{1}{N}\underset{0}{\overset{t}{\int }}\left\{{\beta}_2X(s)Z(s)+p\hbox{'}\delta {I}_H(s)Z(s)+F\left[Y(s),Z(s)\right]\right\} ds\end{array}} $$

## Results

**Fig. 2:**
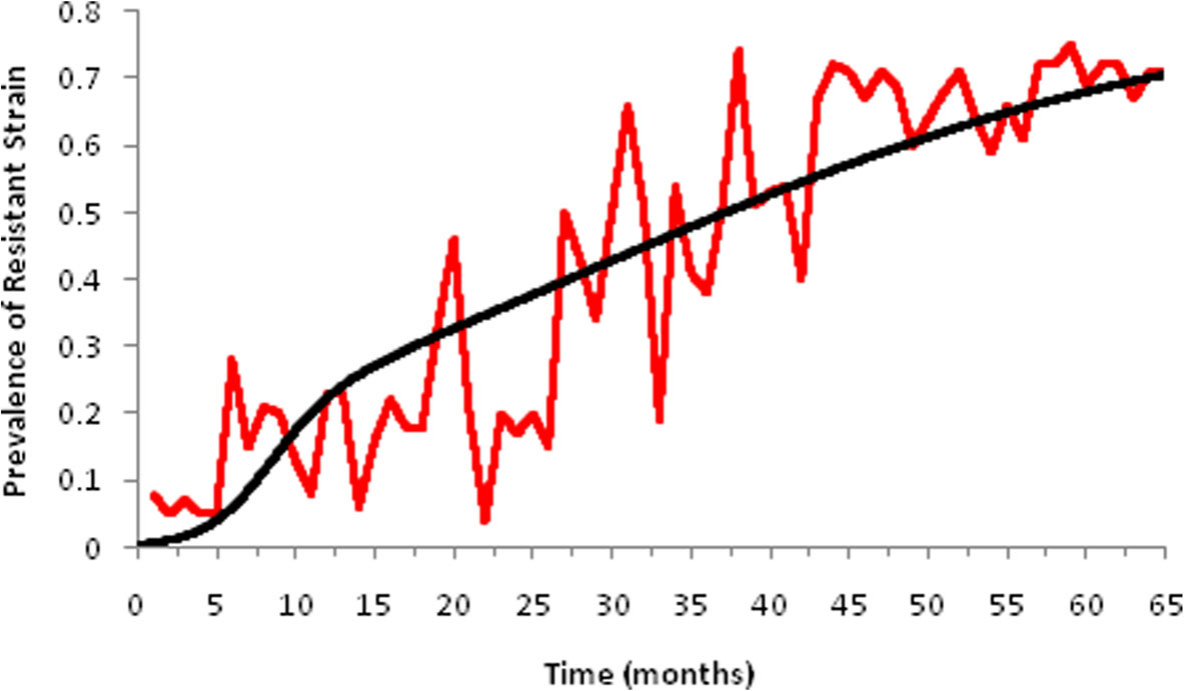
Performance of the model of antibiotic resistance (black line) simulated with parameters as in Table 1, and actual evolution of *Klebsiella pneumoniae* resistance against Amikacin (red line). Real data from Massad, Yang and Lundberg [24]

Model (1) was simulated, first with the antibiotic resistant component only in order to reproduce the results obtained by Massad, Yang and Lundberg [25] with the data from *Klebsiella pneumoniae* strains resistant against the antibiotic Amikacin in the Clinics Hospital of the School of Medicine of the University of São Paulo, Brazil. Results are shown in Fig. 2.

Note that the model tallies the actual data with good accuracy for a fraction of antibiotic treated individuals of 70%. In just 5 years resistance evolved from less than 10% to more than 70%.

**Fig. 3:**
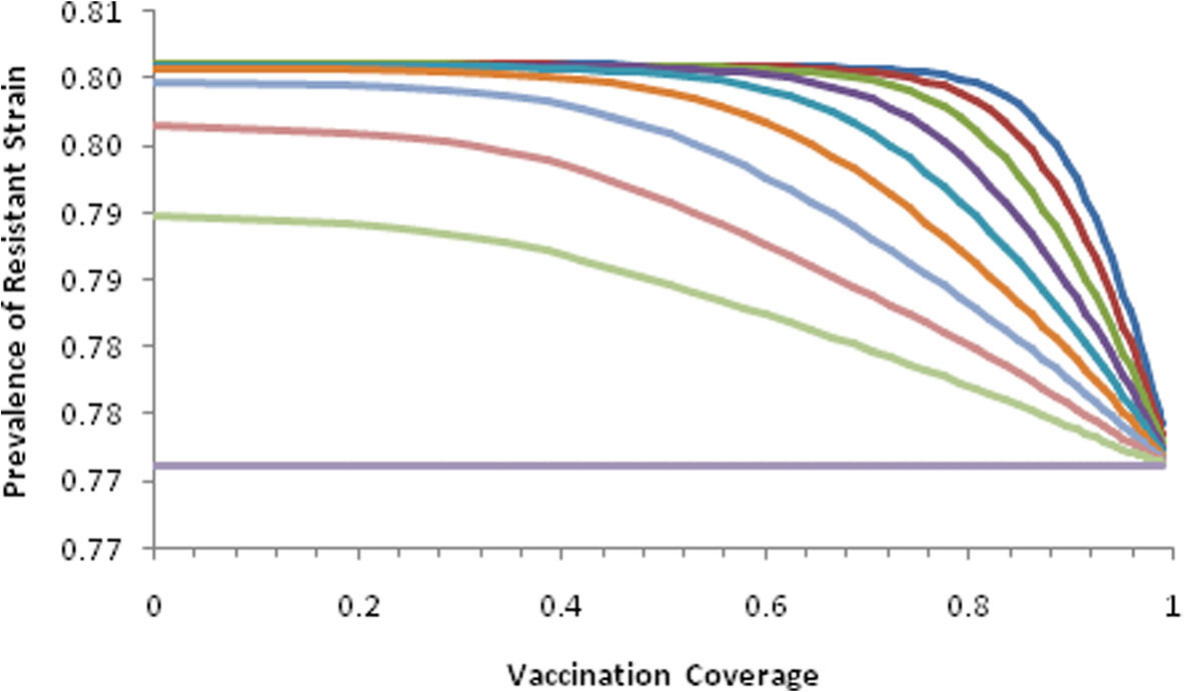
Performance of the complete model of antibiotic resistance and dengue simulated with parameters as in Table 1. Continuous purple line represents the equilibrium after 60 months of treatment in the absence of dengue, that is, the base line evolution of resistance against antibiotics for that specific community. Other lines represent effect of vaccination with several proportions of antibiotic misuse against dengue, varying from 10% (lower light green line) to 50% (upper blue line)

The complete model (1) was then simulated with variables and parameters as in Table 1 in order to estimate the impact of inappropriately treating dengue patients with the antibiotics and the impact of vaccination against dengue on the evolution of antibiotic resistance of dengue-infected individuals mistreated with the same antibiotic (Amikacin) against the same pathogen (*Klebsiella pneumoniae*). The result can be seen in Fig. 3.

Note that the final proportion of resistant bacteria varies in a non-linear fashion with the increase in the proportion of dengue patients inappropriately treated with the antibiotic.

Note that the vaccination coverage necessary to reduce the resistance against the antibiotic in this extreme situation is very high.

## Discussion

In this paper we propose a composite model to test the hypothesis that a hypothetical vaccine against dengue could help to hamper the evolution of resistance against antibiotics due to their misuse in dengue patients. This hypothesis was tested with a composite model combining a previously published model for studying the evolution of antibiotic resistance, with a classical Ross-Macdonald dengue model [26]. The simulation of a real setting involving the overuse of amikacin in patients infected with *K. pneumoniae* in a large hospital in Sao Paulo, Brazil [25] and the inclusion of dengue patients (see Fig. 1) into the bacterial dynamics part of the composite model. The inappropriate use of antibiotics in dengue patients increased the evolution of resistance against these antibiotics in a non-linear fashion. Hence, if 10% of dengue patients were treated with antibiotics, the proportion of bacteria resistant to the drugs would increase from the baseline of 70% to almost 89% and so on as seen in Fig. 3.

The result of the simulation of the impact of the theoretical dengue vaccine also resulted in a highly non-linear decrease in the proportion of resistant bacteria with the increase in the vaccination coverage (Fig. 3). Although it should be expected a reduction in the proportion of resistant bacteria with the reduction of susceptible individuals due to the vaccine, the simulations show that the necessary coverage to result in a significant reduction in the proportion of resistant bacteria is very high. In addition, the higher the proportion of dengue patients mistreated with antibiotics, the higher the necessary vaccination coverage to reduce the antibiotic resistance to base level (Fig. 3).

Our model has several oversimplifications and limitations. Firstly it assumes a homogeneously mixing transmission, both to the bacterial infection and to the dengue infection. The model is deterministic, ignoring eventual stochastic fluctuations in the compartments dynamics. Many of the parameters used in the simulations are not based on empirical observations, although the antibiotic resistant part of the model reproduces a real scenario with a reasonable accuracy. The dengue model is not stratified by serotypes but considers dengue as an all-or-nothing infection. And finally, the theoretical vaccine is assumed to be 100% efficient to all dengue serotypes. Therefore if such a vaccine would be available and if 100% of susceptible people were vaccinated, then no dengue case would occur and no misuse of antibiotic would occur. Considering 50 million dengue cases per year worldwide, considering that between 20 and 40% are mistreated with antibiotics and considering the average cost of one antibiotic course of US$9.91 [27] for each episode of wrongly diagnosed upper respiratory infection (the main cause of antibiotic misuse in dengue patients [2], then it should be expected an economic gain of between US$99,100,000.00 and US$198,200,000.00 per year.

Notwithstanding the above oversimplifications, we think that the composite model served its purposes since it was designed to qualitatively investigate how a hypothetical vaccine could curb the evolution of resistance against antibiotics that is caused by the inappropriate use of these drugs in dengue patients.

It is possible, therefore, based on the results of the simulation of our model that a dengue vaccine would reduce the rate of evolution of antibiotic resistance in a scenario in which dengue patients are inappropriately treated with the drug.

## Conclusion

The use of a dengue vaccine is helpful in reducing the rate of evolution of antibiotic resistance in a scenario of misuse of the antibiotics in dengue patients.

## Data Availability

Please contact author for data request.
